# Supplementary material for: A Critical View of the Application of the APEX Software (Aqueous Photochemistry of Environmentally-Occurring Xenobiotics) to Predict Photoreaction Kinetics in Surface Freshwaters
Source: Molecules. 2019 Dec 18;25(1):9. doi: 10.3390/molecules25010009 (PMC7017383; doi:10.3390/molecules25010009)
Supplement: Supplementary file 1 [file molecules-25-00009-s001.zip › 3.2.4_gcc-4.4.0.zip!/3.2.4_gcc-4.4.0/3.2.4_gcc-4.4.0/doc/octave/html/interpreter/Storage-of-Sparse-Matrices.html]

Storage of Sparse Matrices - Untitled


Next: Creating Sparse Matrices,
Up: Basics


---

#### 21.1.1 Storage of Sparse Matrices

It is not strictly speaking necessary for the user to understand how
sparse matrices are stored. However, such an understanding will help
to get an understanding of the size of sparse matrices. Understanding
the storage technique is also necessary for those users wishing to
create their own oct-files.

There are many different means of storing sparse matrix data. What all
of the methods have in common is that they attempt to reduce the complexity
and storage given a-priori knowledge of the particular class of problems
that will be solved. A good summary of the available techniques for storing
sparse matrix is given by Saad ^1^.
With full matrices, knowledge of the point of an element of the matrix
within the matrix is implied by its position in the computers memory.
However, this is not the case for sparse matrices, and so the positions
of the non-zero elements of the matrix must equally be stored.

An obvious way to do this is by storing the elements of the matrix as
triplets, with two elements being their position in the array
(rows and column) and the third being the data itself. This is conceptually
easy to grasp, but requires more storage than is strictly needed.

The storage technique used within Octave is the compressed column
format. In this format the position of each element in a row and the
data are stored as previously. However, if we assume that all elements
in the same column are stored adjacent in the computers memory, then
we only need to store information on the number of non-zero elements
in each column, rather than their positions. Thus assuming that the
matrix has more non-zero elements than there are columns in the
matrix, we win in terms of the amount of memory used.

In fact, the column index contains one more element than the number of
columns, with the first element always being zero. The advantage of
this is a simplification in the code, in that there is no special case
for the first or last columns. A short example, demonstrating this in
C is.

```
       for (j = 0; j < nc; j++)
         for (i = cidx (j); i < cidx(j+1); i++)
            printf ("non-zero element (%i,%i) is %d\n",
     	   ridx(i), j, data(i));
```

A clear understanding might be had by considering an example of how the
above applies to an example matrix. Consider the matrix

```
         1   2   0  0
         0   0   0  3
         0   0   0  4
```

The non-zero elements of this matrix are

```
        (1, 1)  => 1
        (1, 2)  => 2
        (2, 4)  => 3
        (3, 4)  => 4
```

This will be stored as three vectors cidx, ridx and data,
representing the column indexing, row indexing and data respectively. The
contents of these three vectors for the above matrix will be

```
       cidx = [0, 1, 2, 2, 4]
       ridx = [0, 0, 1, 2]
       data = [1, 2, 3, 4]
```

Note that this is the representation of these elements with the first row
and column assumed to start at zero, while in Octave itself the row and
column indexing starts at one. Thus the number of elements in the
i-th column is given by cidx `(`i `+ 1) -` cidx `(`i`)`.

Although Octave uses a compressed column format, it should be noted
that compressed row formats are equally possible. However, in the
context of mixed operations between mixed sparse and dense matrices,
it makes sense that the elements of the sparse matrices are in the
same order as the dense matrices. Octave stores dense matrices in
column major ordering, and so sparse matrices are equally stored in
this manner.

A further constraint on the sparse matrix storage used by Octave is that
all elements in the rows are stored in increasing order of their row
index, which makes certain operations faster. However, it imposes
the need to sort the elements on the creation of sparse matrices. Having
disordered elements is potentially an advantage in that it makes operations
such as concatenating two sparse matrices together easier and faster, however
it adds complexity and speed problems elsewhere.

---

#### Footnotes

[1] Youcef Saad "SPARSKIT: A basic toolkit
for sparse matrix computation", 1994,
http://www-users.cs.umn.edu/~saad/software/SPARSKIT/paper.ps

---
